# Deployment of Municipal Solid Wastes as a Substitute Growing Medium Component in Marigold and Basil Seedlings Production

**DOI:** 10.1100/2012/285874

**Published:** 2012-01-11

**Authors:** Nikos Tzortzakis, Sofia Gouma, Costas Paterakis, Thrassyvoulos Manios

**Affiliations:** ^1^Department of Organic Greenhouse Crops and Floriculture, School of Agricultural Technology, Technological Educational Institute of Crete, 71004 Heraklion, Greece; ^2^Inter-Municipal Enterprise for the Management of Solid Wastes-IMEMSW, 73100 Chania, Greece

## Abstract

The possible use of municipal solid waste compost (MSWC) in the production of marigold and basil seedlings examined. Six medium prepared from commercial peat (CP) and MSWC (0, 15, 30, 45, 60, and 100% v/v). There was not any plant growth when MSWC used alone (100%). The addition of MSWC in low content (15% and 30%) improved seed emergence for marigold and basil respectively, while greater content revealed opposed impacts. Mean emergence time delayed as MSWC content increased into substrates. Addition of MSWC (especially in content greater than 30%) into CP reduced (from 34 to 64%) plant height, leaf number and stem diameter as a consequence reduced plant fresh weight (plant biomass) for both species. The number of lateral stems decreased (up to 81%) in basil when MSWC added into substrate mixtures. Chlorophyll b content decreased (up to 58%) in substrates with MSWC content greater than 15% or 30% while similar reduction observed in content of Chlorophyll a and total carotenoids for basil with MSWC > 60%. However, Chlorophyll a and total carotenoids content increased as MSWC content increased for marigold. K and Na leaf content increased but P equivalent decreased as MSWC content increased. Nursery-produced basil and marigold seedlings grown in 15% MSWC; displayed quality indices similar to those recorded for conventional mixtures of peat and may act as component substitute.

## 1. Introduction

Transplants, compared with direct sowing, are a more reliable method of ensuring the proper establishment of a range of commercial horticultural crops with great economic value. The production of container-grown flowers and aromatic plants is a highly competitive business; uniform and rapid seed emergence is essential prerequisites to increase yield, quality, and profits in crops. Use of good crop substrates is therefore critical [1].

It is well known that peat, a wide used substrate, is a nonrenewable resource, and diminishing availability is prompting price increases. The extensive use of peat as a substrate has led growers to consider its replacement in the medium to long term [1] with alternative candidates achieving attention. Numerous studies have shown that organic residues such as urban solid wastes, sewage sludge, pruning waste, and even green wastes following composting process can be used with very good results as growth media instead of peat [2, 3]. Additionally, composting has positive environmental impacts towards organic residues. The introduction and interest of compost into nursery plant production increased nowadays. It has been found that mixtures of compost with perlite (20–50% MSWC) may be used as substrates without the need for additional mineral fertilizer [4, 5]. There are, however, certain limitations on some composts use, including the increase in salt content to levels which might affect the growth of sensitive crops, heavy metal toxicity, low overall porosity, and a marked variation in physical/chemical properties [6, 7].

Municipal solid waste compost (MSWC) as an organic soil additive when applied in field trials suggested that it can be used in agricultural production, improving soil physicochemical properties, increasing water retention as well as supply with considerable amount of essential nutrients [8, 9]. However, little information is available regarding the use of MSWC as a peat alternative for nursery production of horticultural crops. Indeed, most studies have focused on ornamental potted plants, woody shrubs, and trees [10]. Each particular compost has to find the best amounts for particular plant growth as there is no one standard growing medium recommended for all container crops under all growing conditions.

A previous study showed that the growth and development of nursery-produced tomato seedlings using a peat and MSWC mixture was similar to that obtained with the standard peat mixture [5]. The present study sought to evaluate the effect of varying the proportion of MSWC mixed with conventional peat substrates, as a growth medium in the nursery production of marigold and basil plants.

## 2. Material and Methods

### 2.1. Seed and Municipal Solid Waste Compost Source

Seeds of marigold (*Tagetes erecta* L. cv Erecta) and basil (*Ocimum basilicum *L. cv sweet basil) were purchased from Agrospito company (Goldsmith seeds Ltd, CA, USA) and Agrimore (Agrimore SA, Thessaloniki, Greece), respectively.

Municipal solid waste compost was punctuated by Inter-Municipal Enterprise for the Management of Solid Wastes (IMEMSW), based in Chania. The compost used was made from the organic fraction of selectively collected urban waste. Following electromagnetic separation, manual sorting and use of an 80 mm trommel screen to remove as many bulking agents as possible, organic material was arranged in piles of 5 m wide of 2.5 m high of 45 m long, which were regularly turned and watered over a 140-day period to ensure appropriate composting conditions (turned windrow system). This material was then passed through a densimetric table and a 15 mm trommel screen to remove the largest particles. The composting procedure lasted for 5-6 months. The 60% of compost consisted of particles with <4 mm size. 

### 2.2. Germination and Plant Growth Studies in Nursery Tests

Marigold and basil seeds were used for nursery tests. A mix of commercial compost peat (Professional peat, Gebr. Brill Substrate GmbH & Co.KG, Georgsdorf, Germany), perlite (Perloflor, Protectivo EPE, Athens, Greece), and MSWC were used to create six treatments which were (% v/v) (1) peat : MSWC (100 : 0) as control, (2) peat : MSWC (85 : 15), (3) peat : MSWC (70 : 30), (4) peat : MSWC (55 : 45), (5) peat : MSWC (40 : 60), and (6) peat : MSWC (0 : 100). In each substrate medium was added 10% of perlite. Perlite physicochemical properties were reported in previous studies [11].

Seeds were sown (0.5 cm depth; 1.0–1.5 cm between seeds in plastic seedling trays (5 seeds per well; 4 wells per replication; 5 replications per treatment, 40 cm^3^ well capacity)) on top of the surface of the media. The experiment was carried out in a completely randomized design in an unheated glasshouse with a north-south orientation at the School of Agricultural Technology at Heraklion, Crete, Greece, located at the latitude of 35′ 35°N, longitude 24′ 02°E, and 8 m altitude (temperature: 25.7 ± 6.8°C max, 15.1 ± 5.1°C min; RH (%): 93.5 ± 1.9 max, 74.8 ± 4.1 min) with alternate-day watering by mist system (initially with 1 min/2 h and then up to 1 min/5 h). Over the growth-period in the nursery, no fertilizer was applied; seedling nutritional requirements were thus met entirely by the substrates. Daily observations were recorded for seed germination (seeds recorded as emerged when the hypocotyls appeared above the surface of substrate medium). After 15 days seedlings were thinned to single plant, maintaining 4-5 cm distance among seedlings. Mean germination time (MGT) was calculated as follows, according to Labouriau [12]:

(1) $$t=\frac{\left(\Sigma ni.ti\right)}{\Sigma n}\;\;\left(\text{days}\right),$$

where *t* is the mean germination time, *ti *is the given time interval, *ni* is the number of germinated seeds during a given time interval, and *n *is the total number of germinated seeds.

After 48 days, seedling growth was assessed by harvesting six individuals/treatment. Seedlings were harvested above substrate, the leaf number and height (cm) per seedling, measured from substrate surface, stem diameter (mm) measured below the cotyledon node, number of stems (for basil), number of flowers (for marigold), upper fresh weight (g), total dry matter content (%), leaf fluoresces, content (*μ*g/g fresh weight) of chlorophyll a (Chla), chlorophyll b (Chlb), and total carotenoids (Car) determined. Leaf elemental analysis for K, Na (photometric), P (spectrophotometric), and N (Kjeldahl) was determined.

### 2.3. Statistical Analysis

The experiment was carried out twice. Percentage data were log-transformed before analysis. Data were tested for normality and then subjected to analysis of variance (ANOVA). Significant differences between mean values were determined using Duncan's Multiple Range test following One-Way ANOVA. Various correlations were also calculated. Statistical analyses were performed using SPSS (SPSS Inc., Chicago, Ill, USA).

## 3. Results and Discussion

### 3.1. Compost Properties

The main physicochemical characteristics of compost (dry weight: dwt) were pH, 7.7; electrical conductivity (EC), 18.2 mS/cm; ashes, 50.6% dwt; organic matter, 49.4% dwt; Carbon, 27.5% dwt; N, 1.9% dwt; and ratio C/N, 7.2, under low limits for heavy metal content. The C/N ratio is widely used as an indicator of the maturity and stability of organic matter. The low values recorded here for the C/N ratio in MSWC suggest that composts were stable and mature. Davidson et al. [13] reported that composts with a C/N ratio of less than 20 are ideal for nursery plant production. Ratios above 30 may be toxic, causing plant death [14].

### 3.2. Seed Germination and Emergence Time *In Vivo*

**Figure 1:**
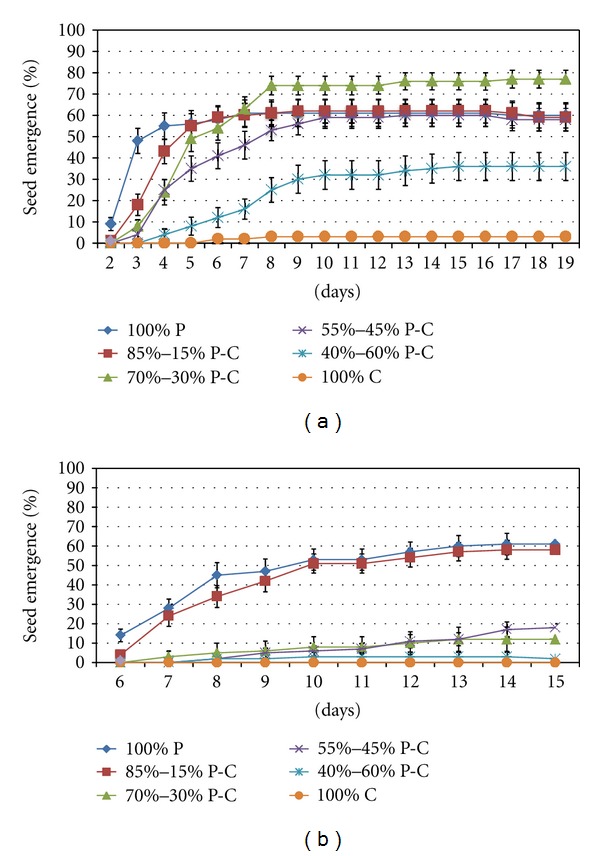
Influence of substrate medium (commercial peat : CP; municipal solid waste compost : MSWC) at different ratios on cumulative seedling emergence of marigold and basil seeds germinated in greenhouse nursery. Values represent mean (±SE) of measurements made on 5 independent replication (4 wells per replication; 5 seeds per well) per treatment.

The first germination was observed after two and six days of sowing for marigold and basil, respectively, while the first true-leaf was emerged after five and nine days, respectively. The ratios of mix when peat and MSWC were used affected seed germination/emergence (Figure 1). Marigold treated with the 70 : 30 CP : MSWC ratios increased (up to 22%) seed emergence comparing with the control (PC) being in accordance with previous studies in melon seedlings [9]. In contrast, high content (>45%) of MSWC into the substrate reduced seed emergence comparing with the control. In basil seedlings, there were no differences with low (15%) MSWC content comparing with control (see Figure 1). Thus, increased MSCW content into the substrate revealed reduction in seed emergence. The worst overall emergence rates were recorded for 100% MSWC, only 3% emergence for marigold by day 8 after which emergence ceased, while no emergence was observed in case of basil.

**Figure 2:**
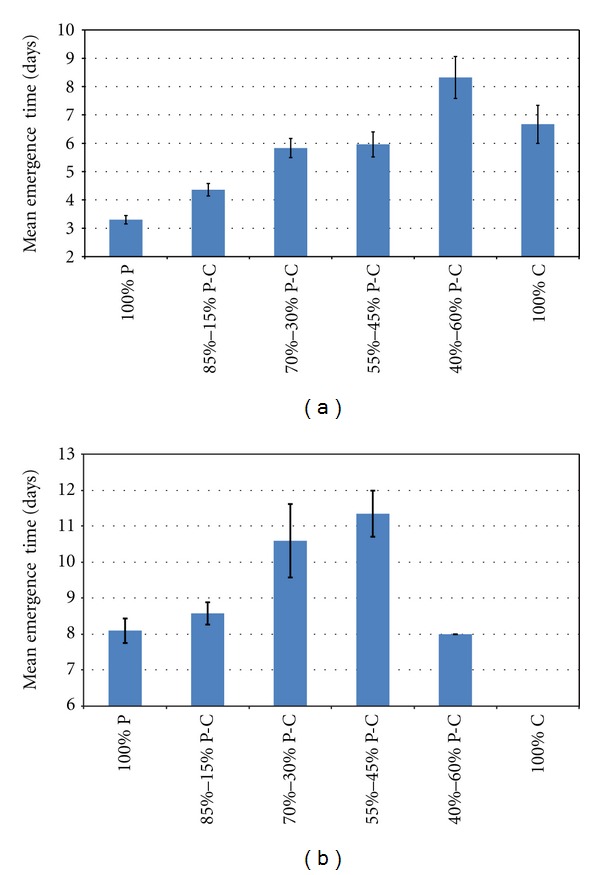
Mean emergence time for marigold and basil in substrate medium (commercial peat : CP; municipal solid waste compost : MSWC) at different ratios under nursery condition. Values represent mean (±SE) of measurements made on 5 replication (4 wells per replication; 5 seeds per well) per treatment.

The ratios of mix when CP and/or MSWC were used affected seed MGT (Figure 2). For marigold seeds, increased MSWC content resulted in MGT reduction. For example, 45% of MSWC revealed 3 days delay in MGT comparing with the control. For basil seeds, MGT delayed as MSWC content increased (up to 45% MSWC content), while in 60% content, MGT did not differ comparing with the control. It is worthwhile to mention that seeds sowed in pure MSWC (100%) did not germinate/emerge at all.

Adding MSWC into the substrate in low content benefits the seed germination/emergence, possible due to the fact that MSWC provided nutritional value as organic material and/or improved substrate medium properties. The stimulation of several presowing treatments (hydropriming, halopriming, osmopriming, thermopriming, solid matrix priming, and biopriming as reported by Ashraf and Foolad [15]) of seed comparing with untreated seeds might be due to altered physiology of embryos and activation of enzymes, so that developmental processes occur more rapidly after sowing [16] and this is possible with the seed germination under MSWC enrichment.

Although there is no single, ideal growth medium for nursery-produced horticultural crops [17], most greenhouse-grown species display better growth at slight acid pH values (5.2–7.0); peat mixtures approached these values but MSWC did not. Like pH levels, the highest initial substrate EC values were recorded for mixtures containing MSWC. Ribeiro and Santos [18] reported that substrates with high EC values reduce water retention, negatively affecting the imbibing process, and may delay seed emergence rates.

### 3.3. Seedling Growth *In Vivo*

Seedling growth failed when 100% MSWC was used as substrate due to seed emergence failure. The highest values for all growth parameters were recorded in substrate without MSWC (control), which in most cases differed significantly from the other substrates (Tables 1 and 2). Analyses of variance showed that the addition (>15% for basil and >30% for marigold) of MSWC in commercial peat significantly reduced leaf number (between 20% and 50%), seedling height (between 20% and 65%), stem diameter (between 27% and 64%), and fresh weight (between 50% and 82%), for basil and marigold seedlings (Table 1) which are in agreement with previous studies in cucumber and melon seedlings [9, 19]. Seedlings dry matter reduced as MSWC content increased into the substrate. Thus, seedlings grown in the MSWC mixtures in high content displayed worse quality and suitability for transplanting, possible due to increased EC and/or alternated medium physicochemical properties. Seedling resistance to transplant stress is directly related to dry matter content, which improves seedling establishment in the soil or growth substrate [20]. In case of basil, stem number reduced as MSWC content increased, and this reduction fluctuated between 57 to 83% comparing with the control treatment, CP. In case of marigold, low content of MSWC into the substrate mixture accelerated the number of flowers produced as well as the number of open flowers (considered open when the yellow colour of petals appeared). 

In marigold seedlings, significant increases in Chla and total carotenoids were observed in substrates with >60% MSWC and >45% MSWC, respectively, while Chlb decreased when the content of MSWC was >45% (Table 3). In basil seedlings, significant increases in Chla and total carotenoids were observed in CP : MSWC (55 : 45) but decreased in substrate CP : MSWC (40 : 60). However, Chlb decreased when the content of MSWC was >30%. No differences were observed in leaf fluoresce among the different substrates. 

Leaf elemental content for marigold revealed that K and Na increase (up to 33% and 83%, resp.) with the addition of MSWC into the substrate, being in agreement with melon seedling production with the same MSWC [9], while P and N content reduced in substrates with >30% and >60% MSWC content, respectively, compared with the control (Table 4). In case of basil, K content increased with the addition of 15–45% MSWC but reduced when MSWC content was greater than 60%. Phosphorus content was reduced as MSWC content increased into the substrate and this might be due to high pH value of MSWC. Nitrogen content decreased in substrates with >60% MSWC content. Thus, considerable nutritive value was marked due to MSWC addition into the substrates. 

MSW compost was found to be an ideal component of mixed-peat substrates for marigold and basil seedlings, provided that it accounts for less than 15% of the mixture. These proportions reduce the negative effects of high pH and EC on seedling growth and provide a seedling comparable to that obtained using standard peat-based mixtures, being in agreement with previous study in melon seedling production [9]. Thus, the mixture of CP (85%) and MSWC (15%) provides an ideal substrate for nursery production of marigold and basil seedlings, yielding quality indices similar to those provided by conventional peat. Similarly, nursery-produced tomato seedlings grown in peat with MSWC (30%) displayed good quality indices [5, 6]. This is in all probability due to a correct balance between nutrient supply from the MSW compost and the physical characteristics of CP, particularly substrate porosity and aeration.

There is a growing public concern about the environmental impact of industrial development and population expansion in recent decades. Improved methods of selective waste collection and compost processing will enable increasingly widespread use of this renewable organic compost, as an alternative to high-quality sphagnum peat, which—because they are nonrenewable—are less available and more expensive for growers.

## Figures and Tables

**Table 1:** Influence of substrate medium (commercial peat : CP; municipal solid waste compost : MSWC) at different ratios on seedling height (cm/plant), number of leaf produced, stem diameter (mm), number of flower produced and opened, and number of lateral shoots on marigold and basil plant grown in greenhouse nursery.

	Marigold					Basil			
	Height	Leaf no.	Stem diameter	Flower no.	Flower open	Height	Leaf no.	Stem diameter	Shoots no.
P-C (100 : 0)	13.57 a^Y^	7.33 a	2.07 a	0.67 c	0.00 c	10.91 a	9.33 a	2.47 a	7.00 a
P-C (85 : 15)	13.87 a	7.33 a	1.65 b	1.83 a	0.50 a	11.58 a	8.33 a	2.49 a	3.00 bc
P-C (70 : 30)	12.82 a	7.33 a	1.99 a	1.67 a	0.50 a	7.25 b	6.51 b	1.65 b	3.33 bc
P-C (55 : 45)	9.6 b	5.67 b	1.41 b	1.00 b	0.17 b	8.75 b	7.50 ab	1.71 b	4.00 b
P-C (40 : 60)	4.8 c	4.50 c	1.51 b	0.50 c	0.00 c	3.96 c	4.66 c	0.89 c	1.33 c
P-C (0 : 100)	—	—	—	—	—	—	—	—	—

^Y^Values (*n* = 6) in columns followed by the same letter are not significantly different *P* < 0.05.

**Table 2:** Influence of substrate medium (commercial peat : CP; municipal solid waste compost : MSWC) at different ratios on seedling fresh weight (g/plant) and dry matter content (%) on marigold and basil plant grown in greenhouse nursery.

	Marigold		Basil	
	Fresh weight	Dry matter	Fresh weight	Dry matter
P-C (100 : 0)	1.92 a^Y^	13.88 b	2.56 a	8.46 b
P-C (85 : 15)	2.38 a	12.37 c	1.98 a	8.45 b
P-C (70 : 30)	2.28 a	18.90 a	1.48 ab	8.45 b
P-C (55 : 45)	0.82 b	11.27 d	1.28 b	8.48 a
P-C (40 : 60)	0.57 c	9.47 d	0.45 c	8.44 b
P-C (0 : 100)	—	—	—	—

^Y^Values (*n* = 6) in columns followed by the same letter are not significantly different, *P* ≤ 0.05.

**Table 3:** Influence of substrate medium (commercial peat : CP; municipal solid waste compost : MSWC) at different ratios on leaf fluoresces, Chlorophyll a (Chla; *μ*g/g fw), Chlorophyll b (Chlb; *μ*g/g fw), and total carotenoids (Car; *μ*g/g fw) on marigold and basil plant grown in greenhouse nursery.

	Marigold				Basil			
	Fluoresce	Chla	Chlb	Car	Fluoresce	Chla	Chlb	Car
P-C (100 : 0)	0.67 a^Y^	53.79 b	67.57 a	19.67 c	0.80 a	57.09 c	29.68 a	28.35 c
P-C (85 : 15)	1.83 a	55.98 b	70.16 a	20.42 c	0.81 a	57.68 bc	25.32 a	30.68 abc
P-C (70 : 30)	1.67 a	61.72 ab	60.36 ab	27.92 bc	0.78 b	58.46 b	16.78 b	30.60 b
P-C (55 : 45)	1.00 a	61.58 ab	50.35 b	30.87 b	0.79 b	58.96 a	15.74 b	31.91 a
P-C (40 : 60)	0.50 a	72.96 a	28.02 c	40.09 a	0.66 ab	38.11 d	18.89 ab	19.10 d
P-C (0 : 100)	—	—	—	—	—	—	—	—

^Y^Values (*n* = 6) in columns followed by the same letter are not significantly different, *P* ≤ 0.05.

**Table 4:** Influence of substrate medium (commercial peat : CP; municipal solid waste compost : MSWC) at different ratios on leaf elemental (K, P, Na, N) concentration (mg/g fresh weight) on marigold and basil seedlings grown in greenhouse nursery. Values represent mean (±SE) of measurements made on 3 replication (3 seedlings mixed per replication) per treatment.

	Marigold				Basil			
	N	K	P	Na	N	K	P	Na
P-C (100 : 0)	10.91 a^Y^	0.177 c	0.020 a	0.015 d	8.58 ab	0.096 b	0.017 a	0.029 b
P-C (85 : 15)	11.02 a	0.231 ab	0.018 ab	0.050 b	7.41 abc	0.120 a	0.013 b	0.036 a
P-C (70 : 30)	12.64 a	0.232 ab	0.016 b	0.136 a	9.06 a	0.084 b	0.009 b	0.029 b
P-C (55 : 45)	11.71 a	0.266 a	0.013 c	0.032 c	8.21 b	0.116 ab	0.008 bc	0.039 a
P-C (40 : 60)	8.93 b	0.228 b	0.017 b	0.122 a	7.33 c	0.048 c	0.005 c	0.017 c
P-C (0 : 100)	—	—	—	—	—	—	—	—

^Y^Values (*n* = 3) in columns followed by the same letter are not significantly different, *P* ≤ 0.05.
